# Three-dimensional motion analysis of ten common Asian sitting positions in daily living and factors affect range of hip motions

**DOI:** 10.1186/s12891-021-04487-z

**Published:** 2021-07-12

**Authors:** Phob Ganokroj, Jirayu Chaowalitwong, Pichitpol Kerdsomnuek, Narumol Sudjai, Pisit Lertwanich, Bavornrat Vanadurongwan

**Affiliations:** Department of Orthopaedic Surgery, Faculty of Medicine Siriraj Hospital, Mahidol University, Bangkok, 2 Prannok Road, Bangkok Noi, Bangkok, 10700 Thailand

**Keywords:** Activities of daily living, Angle, Gender, Hip, Kinematic study, Overweight, Range of motion, Sitting position, Squatting, Three-dimensional motion analysis

## Abstract

**Background:**

Sitting involves many activities of daily life and requires most motion in the hip joint. Asians have more hip flexion and external rotation motions than Westerners owing to cultural and lifestyle differences. Being aware of the normal range of hip motion is essential in clinical practice. Limited research has focused on the hip motions of common sitting positions. The objective was to determine the hip motions of 10 common sitting positions, and to determine whether gender or being overweight affects the range of hip motions.

**Methods:**

An experimental cross-sectional study was conducted to determine hip motions by using a standard, three-dimensional, motion-analysis system. Healthy subjects performed 10 sitting positions during 3 trials. All hip-kinematic data were measured on the dominant leg of each participant, except for the right- and left-monk positions (both hips were analyzed). Density plots were constructed and statistical analyses were performed to detect the differences between groups (male and female; non-overweight and overweight).

**Results:**

The 48 participants comprised 24 males and 24 females. Most were right-leg dominant (45 participants, 93.8%). Of the 22 participants in the overweight group (body mass index ≥23 kg/m^2^), 18 (75%) were male. Squatting showed the highest flexion angle (99.7°, 47.3°–122°). Cross-legged sitting had the highest abduction angle (28.9°, 9.9°–45.7°) and the largest external rotation angle (62°, 37.6°–81.7°). In the female group, there were trends toward a greater flexion angle (4 out of 10 sitting positions) and a smaller abduction angle (6 out of 9 positions), with *P* values < 0.05. As to body weight, the overweight participants had a smaller flexion angle but a greater abduction angle, with 5 out of 9 positions having a *P* value < 0.05. Kinematic data of the transverse plane revealed that the heterogeneity of the rotational angles depended on the sitting position.

**Conclusions:**

This study provided the functional hip motions of common Asian sitting positions. The kinematic data can be utilized in clinical practice as reference values to determine safe positions. Gender and being overweight affected the hip angles in the sagittal and frontal planes.

**Trial registration:**

Number TCTR20181021004, retrospectively registered at the Thai Clinical Trials Registry (http//:www.clinicaltrials.in.th).

## Background

Sitting is an essential human resting position and involves many activities of daily living (ADLs), such as sitting on a chair, using a toilet, and squatting. Sitting requires joint coordination between the hips, knees, and ankles, especially for the hip range of motion (ROM) [1]. Asians have more hip flexion and external rotation ROM angles than Western people. This may result from the differences in their cultures and lifestyles [2, 3]. Squatting requires a high degree of hip movement, such as hip flexion, abduction, and external rotation [1]. Asians commonly squat at least once a day while performing their ADLs or using an Asian-style toilet [1]. [2] Though Westerners do not frequently squat during their ADLs, they use significant hip motion while tying shoelaces, ascending and descending stairs, or lifting objects [4].

It is essential for clinical practitioners to be aware of the normal range of hip motion in the common sitting positions of the ADLs. This basic knowledge can contribute to the development of implants or prostheses that enhance patients’ lifestyles, or to the determination of the safe positions in rehabilitation programs following surgery [1, 5]. For specific hip disorders like femoroacetabular impingement syndrome, this knowledge can assist patients to avoid the aggravating activities (particular sitting positions) that involve a great amount of flexion, adduction, or internal rotation [6]. However, there has been limited research focusing on the hip motions of the common sitting positions that are employed during the ADLs. Moreover, there are differences between the published studies in terms of the sitting positions, measurements, and analytical methods utilized [7–10].. The objective of the present research was to determine the hip ROMs for 10 standard sitting positions used during the ADLs. The authors compared the hip ROMs of healthy male and female subjects for each of a set of predefined sitting positions. The hypothesis was that gender and being overweight would influence the hip ROMs.

## Methods

Before commencement of this research, its protocol was approved by the Institutional Review Board. The work was registered at the Thai Clinical Trials Registry (TCTR20181021004). The study was performed at a motion analysis laboratory at the authors’ facility. The authors enrolled healthy subjects between 18 and 35 years of age. The participants were required to read an information sheet and give written, informed consent. All had no pain or limitations associated with their hip, knee, or ankle motions. The exclusion criteria were: (1) a history of lower extremity fracture; (2) previous lower extremity surgery (hip, knee, or ankle surgery); (3) the presence of any neuromuscular disorder or an impaired balance; (4) pregnancy; (5) a body mass index (BMI) of > 30 kg/m^2^; and (6) an inability to perform all 10 sitting positions due to discomfort.

### Three-dimensional motion analysis (3DMA)

All subjects wore short trousers to enable the secure attachment of reflective markers. Fifteen reflective markers were attached at the following anatomical landmarks: both anterior superior iliac spines (ASIS); the distal lateral femoral condyle; the midpoint between the imaginary line from the greater trochanter to the distal lateral femoral condyle; the distal medial femoral condyle; the midpoint between the imaginary line from the distal lateral femoral condyle to the lateral malleolii; the medial and lateral malleoli; and the sacrum. Eight optoelectronic cameras (Motion Analysis Corp., Santa Rosa, CA, USA) were used to record three-dimensional kinematic data at a sampling rate of 100 Hz. The authors used the Helen Hayes marker model and methods for determining the hip joint center published by Bell et al., which is used widely for measuring kinematics data accurately [11–14]..

Cortex software (Motion Analysis Corp., Santa Rosa, CA, USA) was used to track and process the raw kinematic data filtered using a Butterworth low-pass filter, with a cutoff frequency of 6 Hz. The hip angles were the maximum points achieved while the subjects were sitting still and comfortably. The hip ROM was analyzed using a Cardan rotation sequence of *x*-*y*-*z*. The local *x*-, *y*-, and *z*-axes corresponded to the flexion/extension, abduction/adduction, and internal/external rotation of the hip joint. Kinematic data were set to positive for the flexion, abduction, and external rotation motions. During the measurement, all the skin markers were confirmed for optimal placement.

### Calibrating the system

The calibration was done before recording three-dimensional kinematic data. There was a two-step process of calibration. First, the seed calibration step defined the origin of a coordinate system and orientation of the axes system by using the L-frame placed on the volume of the study. Second, a wand calibration defined the validity of the marker position by using the wand marker waving side to side and up and down through the volume. In this study, the wand length distance was 500 mm. Therefore, the calibration was acceptable when the wand length average was 500 mm, and the deviation was lower than 0.5 mm for accurate three-dimensional data measurement.

### Intervention and measurements

The primary demographic data (age, sex, weight, height, and BMI) were recorded. To determine leg dominance, subjects were asked, “If you kicked a ball at a target, which leg would you use to kick the ball?” [15].

**Fig. 1:**
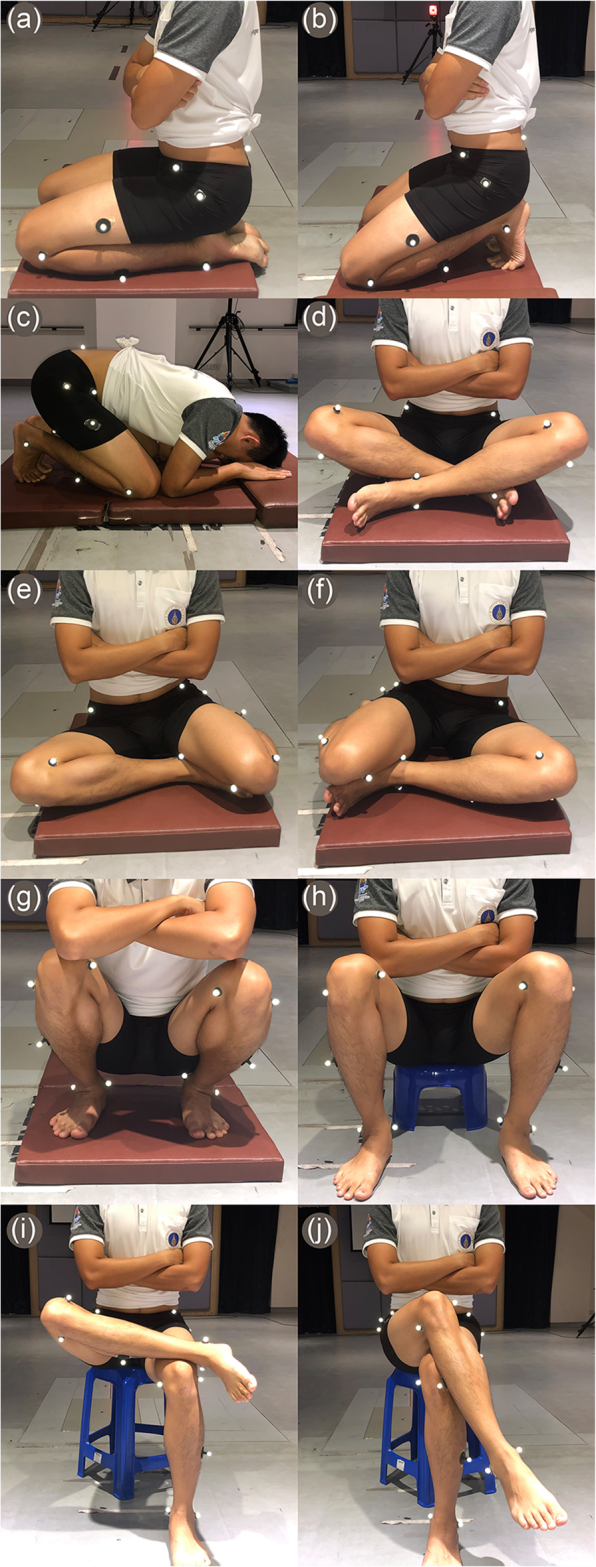
Photographs of the 10 sitting positions: **A**) kneeling plantar-flexed; b) kneeling dorsi-flexed; **C**) benjangkapradit prostration; **D**) cross-legged; **E**) left-monk; **F**) right-monk; **G**) squatting; **H**) sitting on step stool; **I**) figure-four; and **J**) standard leg-cross

All subjects performed the following 10 sitting position: (a) kneeling plantar-flexed; (b) kneeling dorsi-flexed; (c) Benjangkapradit prostration; (d) cross-legged; (e) left-monk; (f) right-monk; (g) squatting; (h) sitting on a step stool; (i) figure-four; and (j) standard leg-cross. (Fig. 1A–J). In Asia, all of the positions are commonly used in the ADLs, while resting, and during social and religious activities.

Before conducting the research, the subjects were shown the correct posture for each of the aforementioned sitting positions and allowed to practice them. The subjects were instructed to hold each sitting position for at least 3 s to enable measurement of angles by the 3DMA equipment. All hip kinematic data were measured on the participant’s dominant leg, except for the right- and left-monk sitting positions (both the left and the right hips were analyzed for those 2 sitting positions). The subjects performed each sitting position three times to calculate the mean and reliability of the measurement, and they were allowed to move their body to feel comfortable between each execution at least one minute break.

During the measurements, the subjects sat on a cushion to not only mitigate discomfort and but to lift the limbs above the floor. An Asian low-style chair (7-in./18-cm height) was used for the sitting-on-a-step-stool position (Fig. 1H). For the figure-four and standard-leg-cross positions, however, a standard plastic chair (18-in./46-cm height) was employed (Fig. 1I–J).

### Statistical analysis

The calculation of the sample size was based on the primary outcome, the ROM of squatting with heels down. It used the minimum, clinically meaningful, difference approximation, which was based on expert opinion. This was estimated to be 10 degrees (i.e., a target difference of 9 degrees). Using a two-sided Student’s t-test, an 80% power to detect a difference in the ROM of squatting with heels down of 9 degrees (SD = 11.0 from a previous study), [7] a 5% level of significance required a sample size of 24 participants per group. A total of 48 subjects were therefore recruited.

The primary outcome was the kinematics of the hip ROMs of 10 sitting positions. Continuous data were described with mean and standard deviation (SD), and median and range; frequency and percentage were used for categorical data. Intraclass correlation coefficient (ICC) was used to present the intrarater reliability of the measurements. The ICCs were determined using a two-way mixed-effects model and single rater (ICC3,1) with absolute agreement. ICC values were interpreted as follows: less than 0.5 as “poor”; 0.50 to 0.75 as “moderate”; 0.75 to 0.90 as “good”; and more than 0.90 as “excellent” [16]. Differences between groups (male and female; and non-overweight and overweight) were analyzed with the Chi-squared test for categorical data, and with Student’s t-test or the Mann–Whitney U test for continuous data. Density plots were constructed, and statistical analyses were performed using the statistical software SPSS for Windows (version 18.0; SPSS Inc., Chicago, IL, USA) and R version 4.0.3 (R Foundation for Statistical Computing, Vienna, Austria). The level of significant difference was set at 0.05.

## Results

The study included 48 participants (24 males and 24 females). Their demographic data is listed in Table 1. The males were taller and heavier, and had higher BMIs, than the females (*P* value < 0.001; Table 1). Most of the participants had a dominant right leg (45 participants; 93.8%). The participants were subdivided into 2 weight groups: non-overweight (BMI < 23 kg/m2) and overweight (BMI ≥ 23 kg/m2), in accordance with the cutoff values for the Asian population [17]. The overweight group comprised 22 participants, 18 (75%) of whom were male (Table 2).

**Table 1:** Demographic data

Characteristic	Total (***n*** = 48)	Male (***n*** = 24)	Female (***n*** = 24)	***P*** value
Age (year)	26.5 ± 4.0	26.2 ± 3.8	26.8 ± 4.2	0.595
Height (cm)	167.1 ± 9.1	174.2 ± 5.9	159.9 ± 5.4	< 0.001^*^
Body weight (kg)	63.1 ± 12.4	73.2 ± 6.8	52.9 ± 7.2	< 0.001^*^
BMI (kg/m^2^)	22.4 ± 3.0	24.1 ± 2.2	20.7 ± 2.6	< 0.001^*^

BMI, body mass index.

The *P* values were for Student’s t-test.

The asterisk (^*^) denotes a statistically significant difference between two groups.

**Table 2:** The number and percentage of participants, stratified by the BMI categories for adult Asian populations

Characteristic	Non-overweight (BMI < 23 kg/m^**2**^) (***n*** = 26)	Overweight (BMI ≥ 23 kg/m^**2**^) (***n*** = 22)	***P*** value
Male (*n* = 24)	6 (25.0%)	18 (75.0%)	< 0.001^*^
Female (*n* = 24)	20 (83.3%)	4 (16.7%)	

BMI, body mass index.

The *P* value was for the Chi-squared test.

The asterisk (^*^) denotes a statistically significant difference between two groups.

**Fig. 2:**
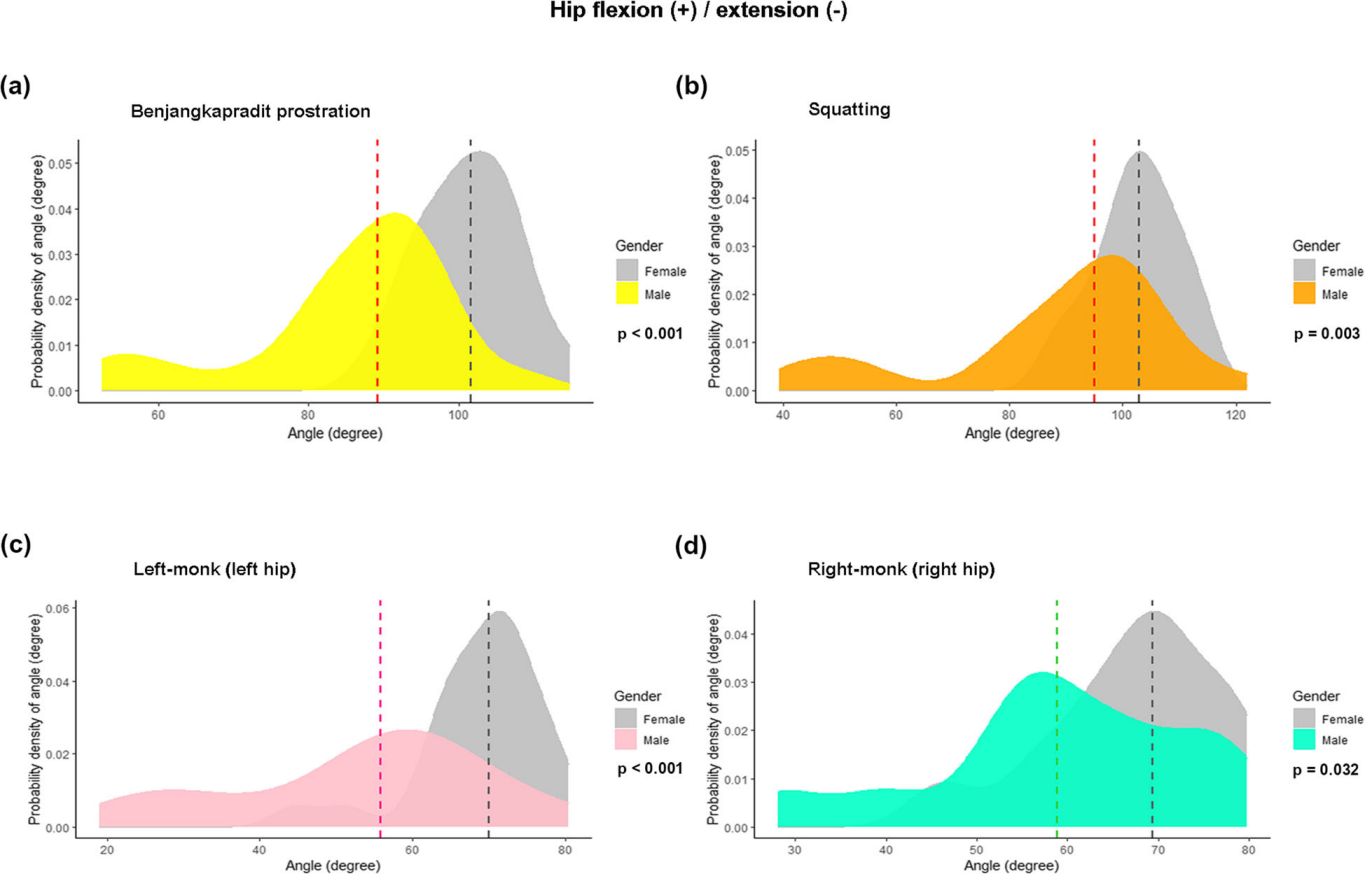
Density plots showing the variations in the joint angles at the hip of 4 sitting positions, stratified by gender: **A**) benjangkapradit prostration; **B**) squatting; **C**) left-monk (left hip); and **D**) right-monk (right hip). The flexion and extension of the hip are the positive and negative values, respectively. The dashed lines signify the median value of the hip joint angle. The *P* values were computed with the Mann–Whitney U test

**Fig. 3:**
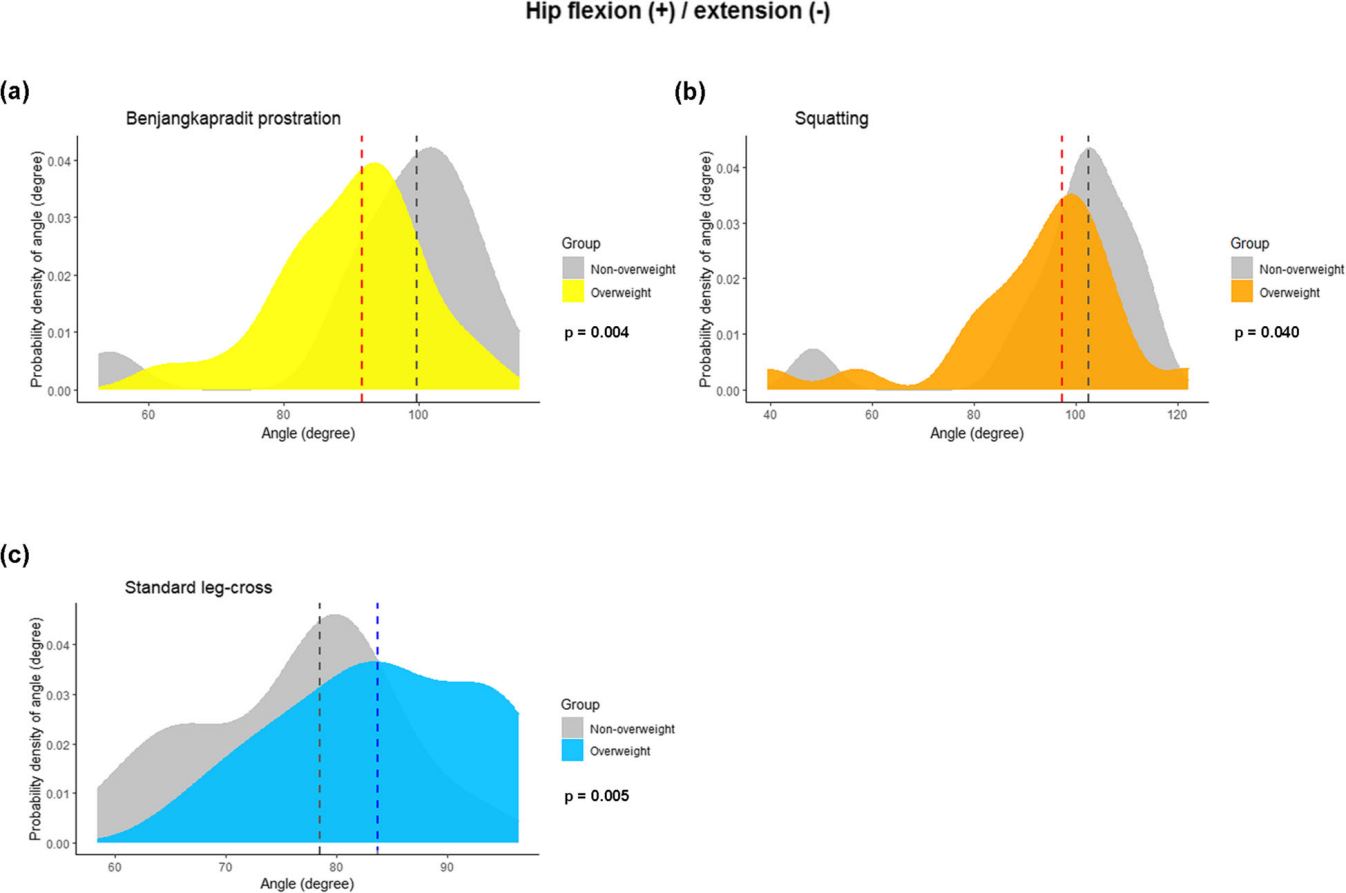
Density plots showing the variations in the joint angles at the hip of 3 sitting positions, stratified by BMI (overweight, BMI ≥ 23 kg/m2): **A**) benjangkapradit prostration; **B**) squatting; and **C**) standard leg-cross. The flexion and extension of the hip are the positive and negative values, respectively. The dashed lines signify the median value of the hip joint angle. The *P* values were computed with the Mann–Whitney U test

The data demonstrated the kinematics of hip angles in all 3 planes (flexion/extension; abduction/adduction; and internal and external rotation) in 10 sitting positions. The measurements were stratified by gender and weight (Tables 3 and 4, respectively). For the hip ROM in the sagittal plane, the hip flexion angle varied between sitting positions. Squatting showed the highest flexion angle (99.7°; 47.3°–122°; Table 3). There was a trend toward a higher flexion angle among the females than the males. Four out of the 10 sitting positions (Benjangkapradit prostration; left-monk position (left hip); right-monk position (right hip); and squatting) had statistically significant differences between the genders (Table 3). Density plots showing the distribution of the flexion angles for those 4 sitting positions, stratified by gender, are presented at Fig. 2. As to BMI, the flexion angles were smaller for the overweight group than the non-overweight group (Table 4 and Fig. 3).

**Table 3:** The measured kinematics of the hips of all participants, stratified by gender

Kinematic data of each sitting position	Total Median degrees (***n*** = 48)	Males Median (range) degrees (***n*** = 24)	Females Median (range) degrees (***n*** = 24)	***P*** value
**Flexion (+) / Extension (−)**				
Kneeling plantar-flexed	67.4	67.1 (38.7 to 79.2)	67.4 (44.1 to 86.1)	0.695
Kneeling dorsi-flexed	51.8	51.9 (29.9 to 60.3)	49.4 (32.4 to 66.7)	0.926
Benjangkapradit prostration	95.3	89.3 (52.5 to 108.7)	101.6 (88.0 to 114.9)	< 0.001^*^
Cross-legged	87.7	87.0 (60.2 to 105.6)	89.7 (73.0 to 105.4)	0.257
Left-monk				
Left hip	64.6	55.9 (19.0 to 80.5)	70.0 (44.4 to 79.2)	< 0.001^*^
Right hip	80.3	81.1 (55.4 to 94.5)	80.2 (63.6 to 93.0)	0.789
Right-monk				
Left hip	79.1	79.4 (60.1 to 96.6)	79.1 (61.9 to 90.5)	0.433
Right hip	65.5	58.9 (28.1 to 79.6)	69.4 (45.5 to 79.8)	0.032^*^
Squatting	99.7	94.9 (47.3 to 122.0)	102.9 (87.1 to 115.4)	0.003^*^
Sitting on a step stool	89.7	88.1 (44.9 to 101.9)	90.9 (74.4 to 101.6)	0.089
Figure-four	83.3	83.1 (58.5 to 104.5)	83.6 (69.6 to 99.7)	0.984
Standard leg-cross	80.0	80.8 (58.4 to 96.4)	80.0 (61.5 to 93.3)	0.386
**Abduction (+) / Adduction (−)**				
Kneeling plantar-flexed	4.3	7.4 (−6.0 to 18.1)	3.5 (−8.4 to 12.9)	0.048^*^
Kneeling dorsi-flexed	7.2	7.8 (−1.1 to 20.4)	5.6 (−4.1 to 13.9)	0.070^*^
Benjangkapradit prostration	4.0	4.3 (−10.3 to 23.7)	3.6 (−7.8 to 24.9)	0.248
Cross-legged	28.9	28.9 (9.9 to 45.7)	29.0 (10.4 to 44.3)	0.773
Left-monk				
Left hip	16.4	23.3 (−1.2 to 55.8)	11.9 (−6.4 to 21.6)	< 0.001^*^
Right hip	8.0	10.5 (−5.0 to 30.7)	6.7 (−4.8 to 21.8)	0.105
Right-monk				
Left hip	7.5	11.9 (−8.5 to 29.4)	4.4 (−7.5 to 14.3)	0.009^*^
Right hip	17.5	22.3 (9.2 to 38.9)	13.7 (−8.4 to 30.7)	0.001^*^
Squatting	8.1	12.5 (−5.1 to 34.3)	5.2 (−6.5 to 20.6)	0.018^*^
Sitting on a step stool	11.5	14.8 (−0.1 to 31.5)	4.3 (−6.0 to 17.8)	< 0.001^*^
Figure-four	16.5	16.9 (2.5 to 35.2)	15.8 (10.1 to 28.7)	0.757
Standard leg-cross	−10.5	−6.9 (−19.9 to 6.0)	−12.5 (−21.7 to 6.7)	0.012^*^
**External rotation (+) / Internal rotation (−)**				
Kneeling plantar-flexed	1.5	1.1 (−8.1 to 20.8)	1.8 (−6.3 to 16.6)	0.734
Kneeling dorsi-flexed	8.8	9.6 (0.4 to 15.9)	6.7 (−8.3 to 22.0)	0.359
Benjangkapradit prostration	2.4	7.6 (−10.8 to 15.5)	−2.0 (− 18.2 to 12.1)	0.001^*^
Cross-legged	62.0	61.2 (37.6 to 73.5)	63.6 (40.7 to 81.7)	0.332
Left-monk				
Left hip	−37.5	−33.7 (− 51.1 to −5.2)	−40.6 (−54.5 to −24.8)	0.014^*^
Right hip	48.3	47.2 (18.7 to 62.5)	50.8 (33.9 to 62.2)	0.034^*^
Right-monk				
Left hip	47.7	47.1 (27.7 to 58.3)	49.4 (42.5 to 58.9)	0.101
Right hip	−37.0	−35.0 (−51.9 to −4.7)	− 37.9 (− 52.3 to −12.0)	0.112
Squatting	− 5.4	− 5.7 (−24.5 to 7.7)	−3.4 (− 20.0 to 5.8)	0.445
Sitting on a step stool	−5.3	−6.9 (− 15.9 to 9.3)	− 3.3 (− 11.0 to 6.8)	0.011^*^
Figure-four	55.2	53.6 (42.9 to 68.6)	56.9 (35.1 to 75.7)	0.578
Standard leg-cross	27.0	32.0 (14.9 to 63.7)	25.0 (2.9 to 48.4)	0.059

The *P* values were for the Mann–Whitney U test.

The asterisk (^*^) denotes a statistically significant difference between two groups.

**Table 4:** The measured kinematics of the hips of all participants, stratified by BMI

Kinematic data of each sitting position	Non-overweight Median (range) degrees (***n*** = 26)	Overweight Median (range) degrees (***n*** = 22)	***P*** value
**Flexion (+) / Extension (−)**			
Kneeling plantar-flexed	67.4 (38.6 to 86.1)	67.1 (53.7 to 79.2)	0.619
Kneeling dorsi-flexed	52.4 (29.9 to 66.7)	50.4 (38.0 to 60.3)	0.346
Benjangkapradit prostration	99.8 (52.5 to 114.9)	91.6 (61.9 to 108.7)	0.004^*^
Cross-legged	85.9 (60.2 to 105.4)	92.0 (62.8 to 105.6)	0.207
Left monk			
Left hip	67.9 (19.0 to 79.2)	63.2 (24.3 to 80.5)	0.077
Right hip	79.8 (55.4 to 93.0)	81.8 (66.4 to 94.5)	0.116
Right monk			
Left hip	78.8 (60.1 to 90.5)	79.8 (67.7 to 96.6)	0.185
Right hip	66.9 (28.1 to 79.8)	63.7 (39.1 to 79.6)	0.649
Squatting	102.5 (47.3 to 115.4)	97.2 (48.7 to 122.0)	0.040^*^
Sitting on a step stool	90.5 (55.5 to 101.6)	88.1 (44.9 to 101.9)	0.242
Figure-four	80.3 (58.5 to 99.7)	83.9 (72.5 to 104.5)	0.166
Standard leg-cross	78.5 (58.4 to 93.3)	83.8 (66.8 to 96.4)	0.005^*^
**Abduction (+) / Adduction (−)**			
Kneeling plantar-flexed	3.2 (−8.4 to 12.9)	6.8 (−6.0 to 18.1)	0.023^*^
Kneeling dorsi-flexed	6.4 (−4.1 to 14.9)	7.8 (−1.1 to 20.4)	0.084
Benjangkapradit prostration	2.6 (−7.8 to 13.7)	6.3 (− 10.3 to 24.9)	0.054
Cross-legged	29.9 (10.4 to 44.3)	28.4 (9.9 to 45.7)	0.619
Left-monk			
Left hip	15.1 (−6.4 to 44.7)	22.8 (−3.7 to 55.8)	0.043^*^
Right hip	6.6 (−4.8 to 21.8)	16.1 (−5.0 to 30.7)	0.024^*^
Right-monk			
Left hip	5.0 (−7.5 to 20.6)	11.2 (−8.5 to 29.4)	0.113
Right hip	14.4 (−8.4 to 37.8)	20.2 (1.8 to 38.9)	0.034^*^
Squatting	7.6 (−6.5 to 24.2)	11.8 (−5.1 to 34.3)	0.475
Sitting on a step stool	6.4 (−6.0 to 23.1)	14.6 (−3.4 to 31.5)	0.018^*^
Figure-four	15.8 (10.1 to 28.7)	17.9 (2.5 to 35.2)	0.396
Standard leg-cross	−13.7 (−21.7 to 3.9)	−6.0 (−19.9 to 6.7)	< 0.001^*^
**External rotation (+) / Internal rotation (−)**			
Kneeling plantar-flexed	1.19 (−6.3 to 16.63)	1.8 (−8.1 to 20.8)	0.627
Kneeling dorsi-flexed	6.7 (−8.3 to 22.0)	9.9 (1.8 to 17.9)	0.136
Benjangkapradit prostration	1.2 (−12.7 to 12.1)	6.7 (−18.2 to 15.5)	0.301
Cross-legged	64.4 (37.6 to 81.7)	57.8 (40.7 to 80.5)	0.051
Left-monk			
Left hip	−36.6 (−54.5 to −15.5)	− 37.5 (−53.4 to − 5.2)	0.860
Right hip	50.4 (33.9 to 62.2)	47.2 (18.7 to 62.5)	0.166
Right-monk			
Left hip	49.5 (37.1 to 58.9)	46.2 (27.7 to 54.1)	0.036^*^
Right hip	−37.0 (−52.3 to −9.9)	− 36.3 (− 51.9 to −4.7)	0.852
Squatting	−3.4 (−23.7 to 7.7)	−6.3 (−24.5 to 4.0)	0.128
Sitting on a step stool	−4.6 (− 15.9 to 6.8)	−7.6 (− 15.7 to 9.3)	0.069
Figure-four	59.1 (42.9 to 68.9)	51.6 (35.1 to 75.7)	0.072
Standard leg-cross	27.4 (2.9 to 45.2)	26.7 (12.9 to 63.7)	0.555

The *P* values were for the Mann–Whitney U test.

The asterisk (^*^) denotes a statistically significant difference between two groups.

**Fig. 4:**
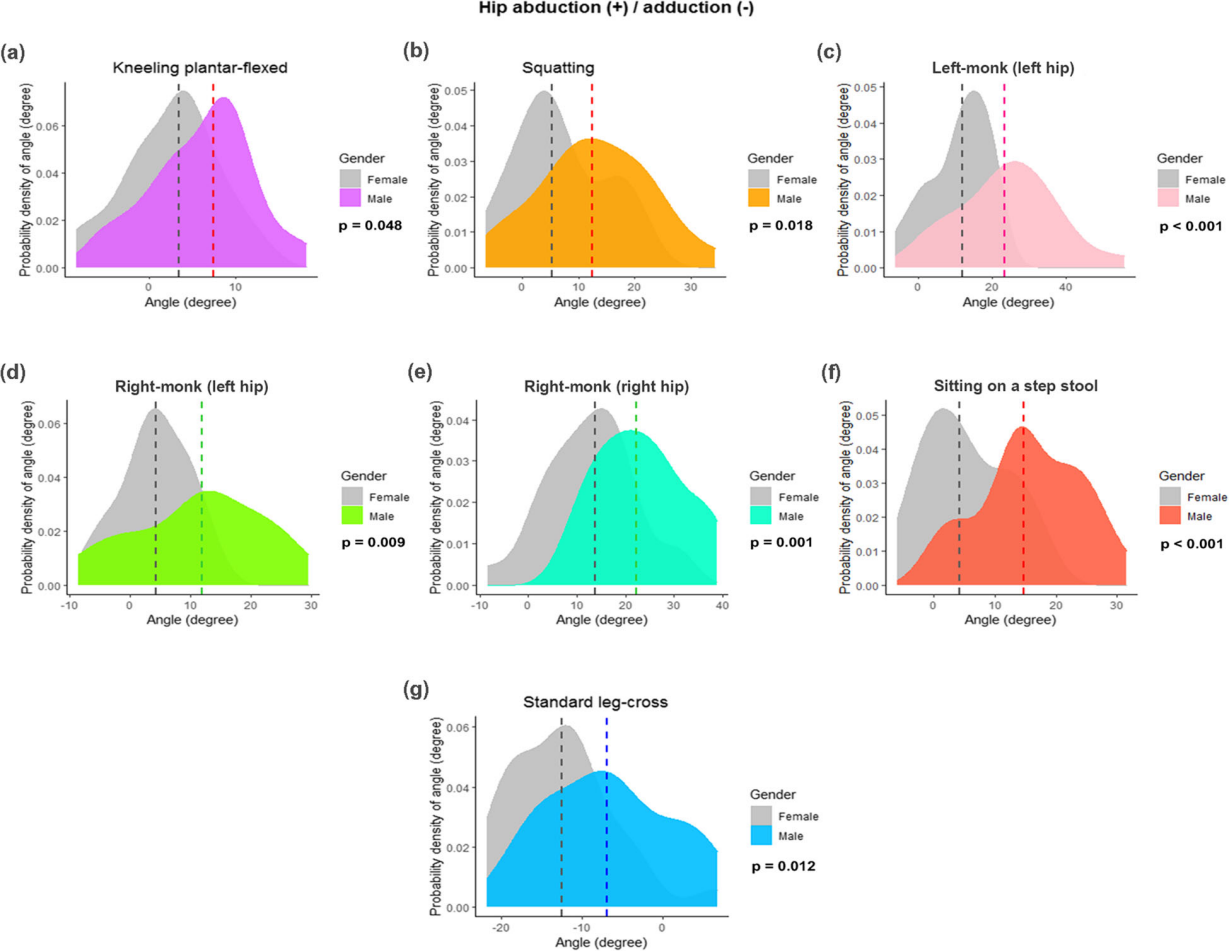
Density plots showing the variations in the joint angles at the hip of 7 sitting positions, stratified by gender: **A**) kneeling plantar-flexed; **B**) squatting; **C**) left-monk (left hip); **D**) right-monk (left hip); **E**) right-monk (right hip); **F**) sitting on a step stool; and **G**) standard leg-cross. The abduction and adduction of the hip are positive and negative values, respectively. The dashed lines signify the median value of the hip joint angle. The *P* values were computed with the Mann–Whitney U test

**Fig. 5:**
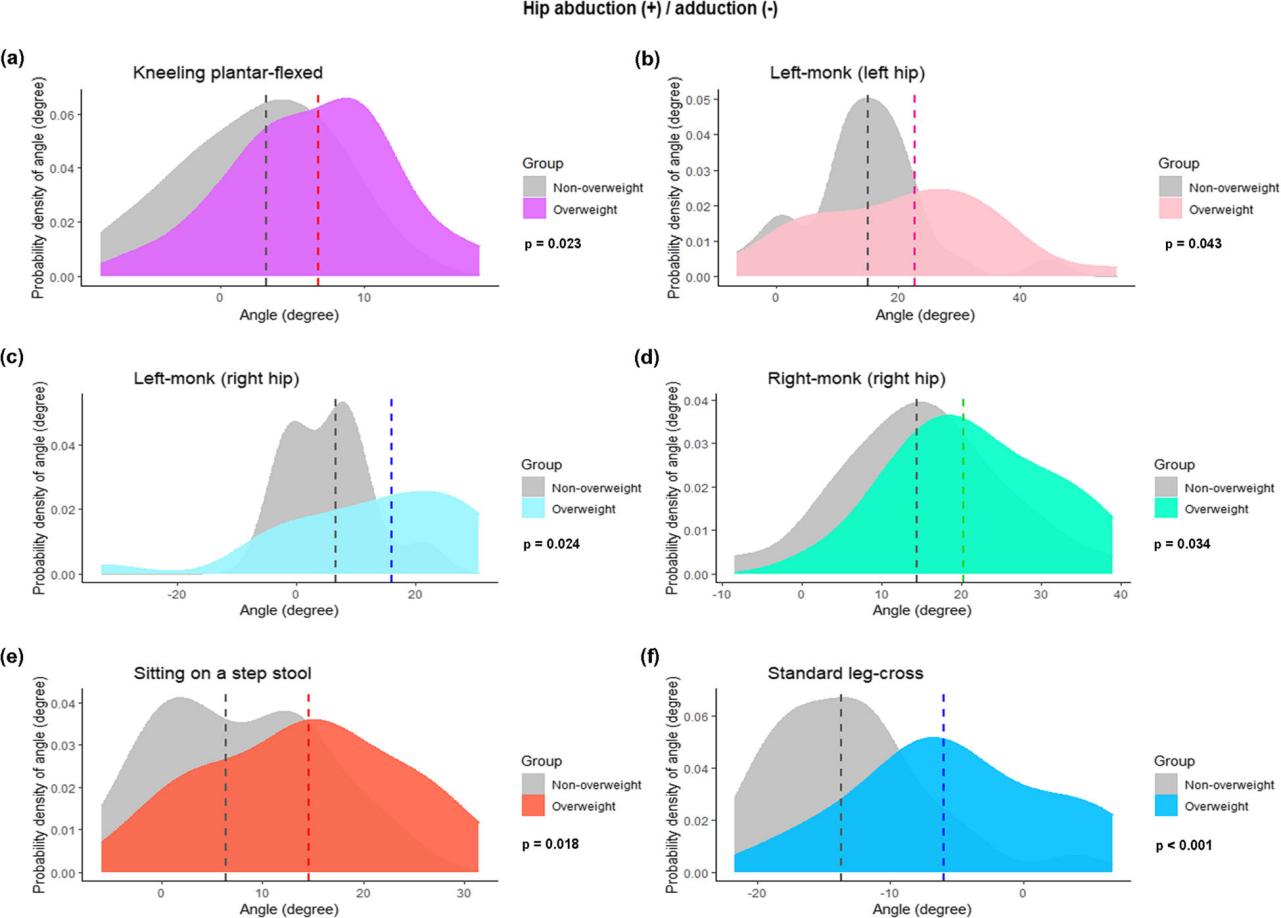
Density plots showing the variations in the joint angles at the hip of 6 sitting positions, stratified by BMI (overweight, BMI ≥ 23 kg/m2): **A**) kneeling plantar flexed; **B**) left-monk (left hip); **C**) left-monk (right-hip); **D**) right-monk (right hip); **E**) sitting on a step stool; and **F**) standard leg-cross. The abduction and adduction of the hip are positive and negative values, respectively. The dashed lines signify the median value of the hip joint angle. The *P* values were computed with the Mann–Whitney U test

In the frontal plane, there were more hip abduction angles in males than in females, with 6 out of 9 sitting positions demonstrating a statistically significant difference (Table 3 and Figs. 4A–F). There was also a trend for more abduction angles in the overweight group: 5 out of 9 sitting positions had a *P* value < 0.05 (Table 4 and Fig. 5). The cross-legged position had the highest abduction angle (28.9°; 9.9°–45.7°). The standard leg-cross position was the only one that represented hip adduction of 10.5° (− 6° to 21.7°). Significantly more adduction angles were found in the female group than the non-overweight group, with a *P* value < 0.05 (Figs. 4G and 5F and G).

**Fig. 6:**
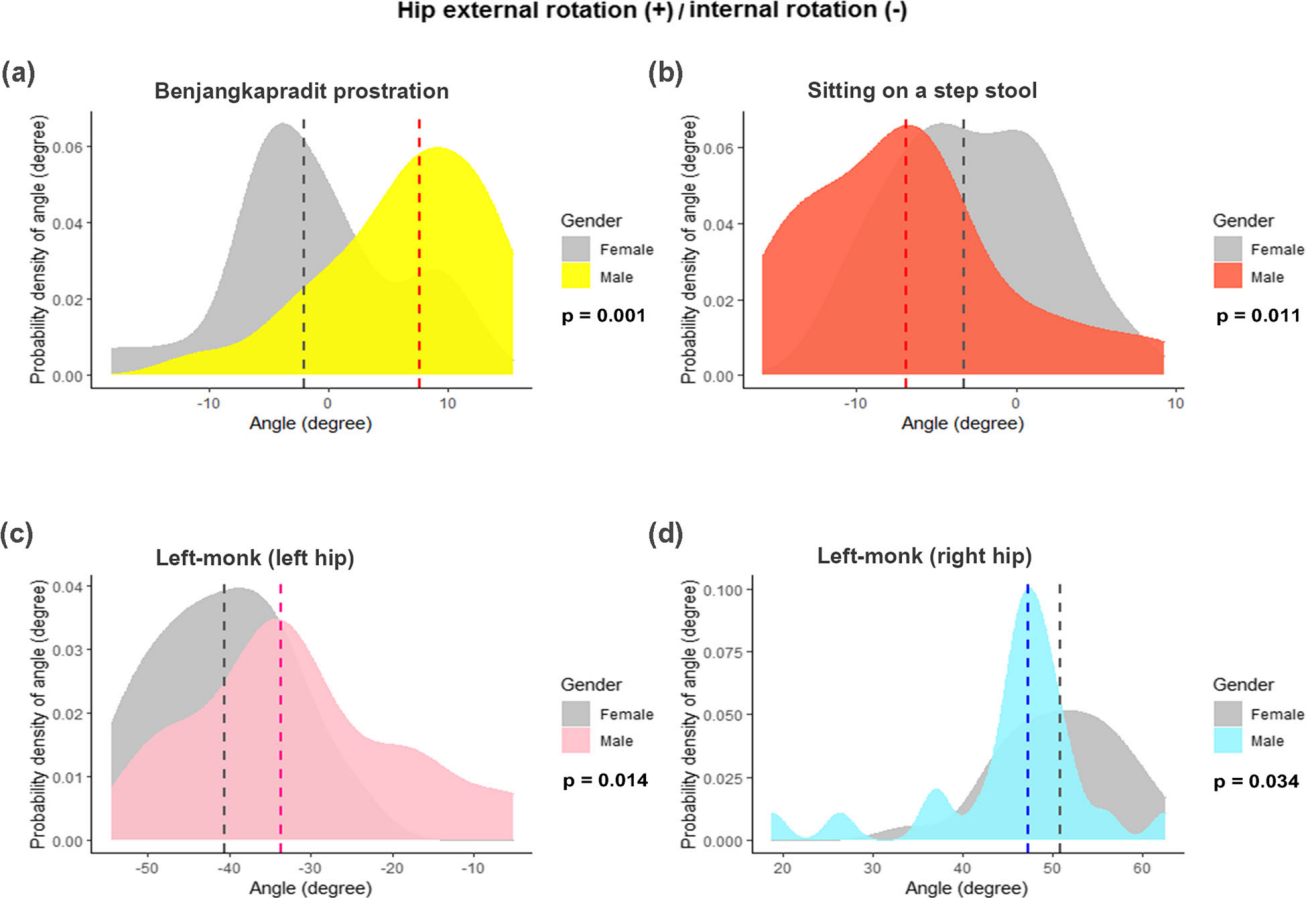
Density plots showing the variations in the joint angles at the hip of 4 sitting positions, stratified by gender: **A**) benjangkapradit prostration; **B**) sitting on a step stool; **C**) left-monk (left hip); and **D**) left-monk (right hip). The external and internal rotations of the hip are the positive and negative values, respectively. The dashed lines signify the median value of the hip joint angle. The *P* values were computed with the Mann–Whitney U test

**Fig. 7:**
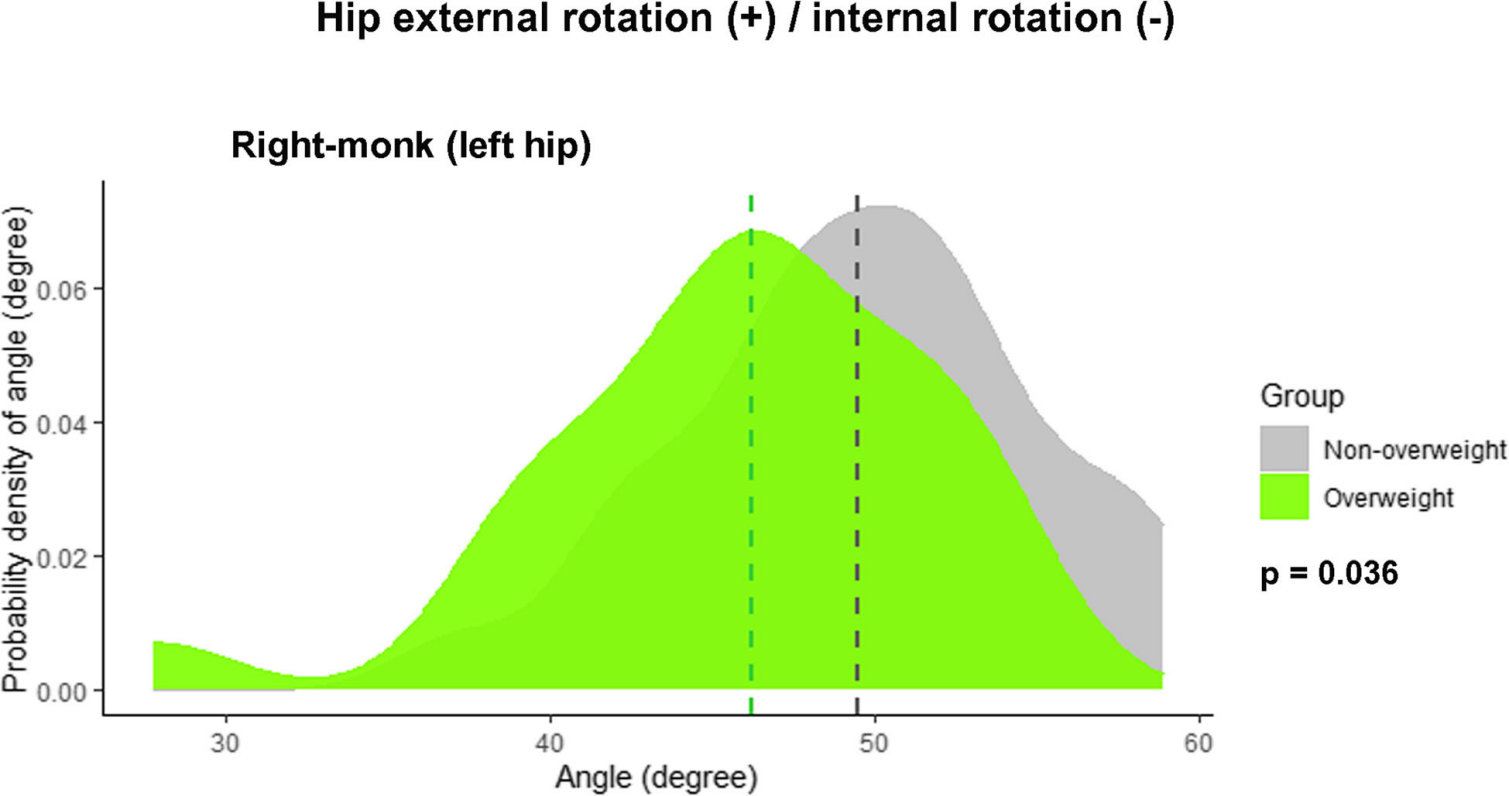
Density plots showing the variations in the joint angles at the hip for a right-monk (left hip) position, stratified by BMI (overweight, BMI ≥ 23 kg/m2). The external and internal rotations of the hip are the positive and negative values, respectively. The dashed lines signify the median value of the hip joint angle. The *P* values were computed with the Mann–Whitney U test

The kinematic data of the transverse plane revealed that the heterogeneity of the rotational angles depended on the sitting position (Tables 3 and 4, and Fig. 6). The right-monk (left hip) position showed more external rotation angles in the non-overweight group (Fig. 7). The cross-legged position had the highest external rotation angle (62°; 37.6°–81.7°), while the left-monk position (left hip) had the highest internal rotation angle (37.5°; 5.2°–54.5°; Tables 3 and 4). Intrarater reliability of measurements in this study showed good to excellent, with ICC values ranging from 0.82 to 0.98.

## Discussion

This study focused on the kinematic data of the hip motions of 10, common, Asian sitting positions. The significant finding was that, of the 10 positions, squatting showed the highest flexion angle (99.7°; 47.3°–122°). Our finding was similar to that of research on Indian subjects. That work investigated 5 sitting positions: squatting with the heels down; squatting with the heels up; kneeling dorsi-flexed; kneeling plantar-flexed; and cross-legged. The Indian study found that squatting with the heels down produced the maximum hip flexion angle (95.4° ± 27°) [7]. Squatting is best defined as the position where the foot contacts the ground in a way that brings the body down over the foot, requiring maximum hip flexion. Many ADLs require the squat position, such as toileting (especially with Asian-style toilets) [1]. In Western countries, the basic squat and chair squat are functional, multi-joint exercises that generate maximum hip flexion (particularly the chair squat) [18].

In this study, the cross-legged sitting position had the highest abduction angle (28.9°; 9.9°–45.7°) and external rotation angle (62°; 37.6°–81.7°). The cross-legged position is also known as the “crossed-legged tailor” position and the “Buddha” position. In Thailand, it is commonly used for resting, eating on a floor, or leisure activities such as yoga [1]. Moreover, the ability to sit cross-legged is one of the functional outcomes employed to evaluate patients after a total hip arthroplasty [19]. The study on the Indian population performing the cross-legged sitting posture found a mean abduction angle of 39° (19°–57°) and a mean external rotation angle of 49° (42°–58°) [10]. That work found more hip abduction angles and fewer external rotation angles than our study. However, the Indian research used a simple manual goniometer to measure angles; those values would be less accurate than ones obtained with 3DMA equipment, especially for rotational angles [20]. In comparison, a kinematic study of Chinese people found a median abduction angle of 12.7° (1.3°–32.7°) and a median external rotation angle of 2.3° (− 11.9°–36.4°). Those values are much lower than the corresponding ones from our study [8]. The Chinese study subjects were instructed to sit cross-legged with a foam cushion only underneath their buttocks, not at the leg and foot areas. However, that particular way of sitting cross-legged is not commonly used for the performance of Asian-style ADLs. It might also lower the hip abduction and external rotation angles.

The authors of the current investigation found that the hip ROMs were associated with both gender and being overweight. There were more flexion angles (4 out of 10) and fewer abduction angles (6 out of 9) in the female group, with a *P* value < 0.05. However, there was no direct relationship between gender and rotational angles. The hip rotational ROM depended on the sitting position. In the case of the overweight subjects, there were fewer hip flexion angles and more hip abduction angles, with 5 out of 9 sitting positions having a *P* value < 0.05. The kinematic data of the transverse plane showed the heterogeneity of the rotational angles depended on the sitting position. In a kinematic study, Huffman et al. investigated the effects of higher BMI on hip ROM [21]. They demonstrated that, during the sitting and sit-to-stand postures, there was a greater increase in the peak abduction angle for their high-BMI group than for their normal subjects [21]. We believe that the lower hip flexion ROM of the overweight group in the current research may have been connected with a higher level of posterior thigh tissue (indicated by the greater thigh circumference of those subjects) as well as movement while changing position. To our knowledge, no study has previously focused on the associations between either gender or weight group and the hip ROMs during the sitting positions used for the ADLs. However, one kinematic study compared the ROMs of young adults and the elderly while kneeling [9]. That work found no apparent differences in the knee and ankle joints of the 2 groups. Nevertheless, the research did find a higher maximum hip flexion angle for the elderly than the young adults (100.5° and 67.5°, respectively) [9]. In the current study, the authors recruited healthy adults aged under 35 years. The age factor would therefore have been most unlikely to affect the main findings of our study. The authors suggest that all factors–gender, weight, and age–should be considered while determining the hip ROM for each sitting position used for the ADLs.

The increase in knowledge gained through kinematic data studies benefits various professional fields. For physiotherapists and prosthetists, gaining a better understanding of these functional motions might aid the development of prosthetic designs that meet the functional needs of patients (especially those in non-Western countries). Orthopedists can adapt the knowledge to treat patients both conservatively and surgically. Activity modification and the avoidance of aggravating activities are key to the conservative treatment of hip disorders. Labral tears and intra-articular hip pathologies are often associated with groin pain that is exacerbated by flexion and rotatory hip movements [22]. The symptoms experienced by femoroacetabular impingement syndrome patients are aggravated in the flexed, adducted, and internal rotated positions [23]. Patients diagnosed with subspine hip impingement suffer severe pain with hip flexion angles greater than 90 degrees [24]. Being aware of the reference values of the hip motions of each common sitting position will help with the education of patients who have hip disorders. In turn, it will assist them to minimize or prevent symptoms.

In order to apply kinematic data to patients who had undergone a total hip arthroplasty (THR), Miki et al. investigated the anatomical hip ROMs after THR using a navigation system [25]. They found a wide range of passive hip ROMs intraoperatively: 113° of flexion, 46° of abduction, 75° of internal rotation, and 36° of external rotation [25]. That research team then studied the patients immediately after their THRs to determine the effects, if any, of the anesthesia, muscle relaxants, surgical techniques, and implant designs that had been utilized. However, their reference values should be cautiously applied in clinical practice. Four sitting positions in the current study (cross-legged, figure-four, left-monk, and right- monk) demonstrated external rotation angles that were higher than the reference values determined by Miki and colleagues [25]. The differences between measuring methods and subjects may be the reasons why there are more external rotation angles in our study. Hence, patients who undergo THR should be cautioned about immediately using these 4 positions. Generally, surgeons will know the safe and stable hip ROMs in the operative theater. Understanding these data could prevent dangers, such as impingement or dislocation after sitting during the ADLs. Physical medicine and rehabilitation physicians might apply these data to design patients’ postoperative protocols and prevent adverse events.

This study had several limitations. Firstly, there was a wide variety of ways in which the 10 sitting postures were executed and achieved. These might be the main reason for the high variety of hip angles in the study. The authors attempted to reduce variations by demonstrating the correct posture for each sitting position and allowing the participants to practice them before the trial. The maximum angle achieved for each sitting position was measured; this is where the use of a 3DMA system is superior to a static measuring device like a goniometer. Secondly, the accuracy of the measurement was the primary concern of this study. The authors chose the reflective skin markers and firmly attached them to the participant’s anatomical landmarks. Using pins or other devices as the bone markers would be more accurate than the skin markers. However, those devices are more suitable for cadaveric studies and could not be used in routine clinical practice. The authors try to solve these problems by measuring each sitting position three times each and ensuring that all markers were perfectly set in the anatomical landmarks during the angle calculation. Moreover, the authors calculated the intra-rater reliability and showed good to excellent in this study. Thirdly, all subjects were young and healthy. These data do not cover elderly patients nor those with lower extremity disorders. Finally, the study regarded the hip angles as the intersegment angle between the pelvis axis and the thigh. No data were collected on spinopelvic parameters that might affect the hip joint while changing position. However, as all subjects in the study were healthy volunteers, the authors believe that all would have normal spinopelvic mobility. The pelvic tilt is an interesting variable for further research as it might affect the sitting posture of patients with pathologies of the spine and/or hip.

## Conclusions

This study provided the functional hip motions of common Asian sitting positions. These kinematic data can be applied in clinical practice as reference values to determine safe positions. Gender and being overweight affected the hip angles in both the sagittal and frontal planes.

## Data Availability

The datasets used and/or analyzed during the current study are available from the corresponding author on reasonable request.

## Abbreviations

BMI: body mass index
ADLs: activities in daily life
ROM: range of motion
3DMA: three-dimensional motion analysis
ASIS: anterior superior iliac spine
SD: standard deviation
THR: total hip replacement
